# Secoisolariciresinol Diglucoside Alleviates LPS‐Induced Acute Lung Injury by Inhibiting the NF‐κB/NLRP3 Signaling Pathway

**DOI:** 10.1002/ddr.70285

**Published:** 2026-04-27

**Authors:** Yuanyuan Zhong, Yang Zou, Zhen Qu, Kai Zhou, Huijuan Liu, Jiali Yang, Chengzhong Tang, Yuqiang Xu, Zhen Wang

**Affiliations:** ^1^ Department of Gerontology People's Hospital of Leshan Leshan Sichuan China; ^2^ Department of Otolaryngology People's Hospital of Leshan Leshan Sichuan China; ^3^ Department of Gastroenterology People's Hospital of Leshan Leshan Sichuan China; ^4^ Department of Gastroenterology General Hospital of Ningxia Medical University Yinchuan China; ^5^ Departement of Otolaryngology and Head and Neck Surgery People's Hospital of Jiangyou Jiangyou Sichuan China

**Keywords:** acute lung injury, inflammation, NF‐κB, NLRP3, SDG

## Abstract

Acute lung injury (ALI) and its more severe form, acute respiratory distress syndrome (ARDS), are life‐threatening pulmonary disorders with high mortality rates, and effective treatments are currently lacking. Secoisolariciresinol diglucoside (SDG), a plant lignan derived from flaxseed, possesses anti‐inflammatory and antioxidative activities. However, the underlying mechanisms by which SDG ameliorates ALI remain incompletely understood. This study aimed to investigate whether SDG alleviates ALI by modulating the NF‐κB/NLRP3 signaling pathway. For the in vivo study, ALI was induced in mice through intranasal administration of LPS. Key indicators included lung histopathological changes, wet/dry weight ratio (W/D), protein concentration in bronchoalveolar lavage fluid (BALF), oxidative stress markers (MDA, SOD, CAT), the expression of inflammatory cytokines and chemokines (IL‐1β, IL‐18, TNF‐α, CCL2), and the level of NF‐κB/NLRP3 pathway‐related proteins. In vitro experiments using LPS‐stimulated RAW264.7 further explored the effects of SDG on the NF‐κB/NLRP3 pathway. SDG significantly mitigated LPS‐induced lung histopathological damage and nasal mucosal injury, reduced lung W/D ratio and BALF protein, and suppressed oxidative stress. Moreover, SDG downregulated pro‐inflammatory cytokines (IL‐1β, IL‐18, TNF‐α) and macrophage infiltration. It also decreased the expression of N‐κB/NLRP3 pathway‐related proteins. In vitro experiments further confirmed that SDG inhibited the NF‐κB/NLRP3 pathway. SDG effectively alleviates LPS‐induced ALI through its antioxidant, anti‐inflammatory, and NF‐κB/NLRP3 pathway‐inhibiting properties, providing experimental evidence for its potential as a therapeutic agent for ALI.

## 1 Introduction

Acute lung injury (ALI) and its severe form, acute respiratory distress syndrome (ARDS), are critical conditions characterized by excessive pulmonary inflammatory responses and disruption of the alveolar‐capillary barrier (Rawal et al. 2018; Esquivel‐Ruiz et al. 2021). Clinically manifested as progressive hypoxemia and respiratory failure, these conditions pose a serious threat to patient survival (Ware and Matthay 2000). Epidemiological data indicate that ALI/ARDS carries a high mortality rate ranging from 34% to 46%, with prognosis closely related to patient comorbidities and underlying diseases (Bellani et al. 2016). Etiologically, lipopolysaccharide (LPS), an endotoxin derived from Gram‐negative bacteria, serves as a major pathogenic factor in ALI development. Recent studies have demonstrated that LPS triggers the release of excessive pro‐inflammatory cytokines (TNF‐α, IL‐1β) from immune cells, leading to a cytokine storm that plays a pivotal role in the pathogenesis of ALI (Dhlamini et al. 2022; Shan et al. 2024). Despite its clinical severity, there remains a lack of effective targeted therapies for ALI/ARDS (Matthay and Zemans 2011). Exploring novel treatment strategies is urgently needed.

The NLRP3 inflammasome plays a pivotal role in the pathogenesis of ALI (Gu et al. 2024). As a critical component of the innate immune system, the NLRP3 inflammasome consists of NLRP3 inflammasome is composed of NLRP3 (nucleotide‐binding domain leucine‐rich repeat [NLR] and pyrin domain containing receptor 3), ASC (apoptosis‐associated speck‐like protein containing a caspase recruitment domain), and procaspase‐1 (Karasawa and Takahashi 2017). Upon stimulation by pathogen‐associated molecular patterns (PAMPs) or damage‐associated molecular patterns (DAMPs), the NLRP3 inflammasome is activated, recruiting and activating caspase‐1. This enzyme cleaves gasdermin D (GSDMD) into C‐terminal (C‐GSDMD) and N‐terminal (N‐GSDMD) fragments, promoting the release of interleukin (IL)‐1β and IL‐18 and inducing pyroptosis and inflammatory cytokine release (Sborgi et al. 2016; Shi et al. 2015; He et al. 2015). This process disrupts the integrity of alveolar epithelial cell membranes, releasing large amounts of damage‐associated molecules that further amplify the inflammatory response (Z. Li et al. 2023). Nuclear factor‐κB (NF‐κB), a transcription factor regulating the expression of various proinflammatory cytokines, acts as an upstream activator of NLRP3 (Bauernfeind et al. 2009). When Toll‐like receptors recognize pathogen signals, they activate NF‐κB via the MyD88/TRIF‐dependent pathway, facilitating its translocation into the nucleus. NF‐κB then directly binds to the promoter region of the NLRP3 gene, upregulating the transcription of NLRP3 and its downstream effector molecules. Therefore, targeted modulation of the NF‐κB/NLRP3 signaling pathway represents a promising therapeutic strategy for ALI.

Secoisolariciresinol diglucoside (SDG) is a plant lignan isolated from flaxseeds. After entering the body, it can be converted into the mammalian lignans enterolactone and enterodiol by the intestinal microbiota, thereby exerting physiological effects (Imran et al. 2015). Extensive studies have confirmed that SDG possesses abundant bioactivities, including anti‐inflammatory, antioxidant, anti‐diabetic, and anti‐tumor effects (Pietrofesa et al. 2022; J. C. Lee et al. 2008; Prasad 2001; D. Li et al. 1999). In terms of its anti‐inflammatory mechanisms, substantial evidence indicates that SDG exerts anti‐inflammatory effects by regulating multiple signaling pathways, such as inhibiting the Akt/IκB/NF‐κB pathway and suppressing the NLRP3 inflammasome (S. Zhang et al. 2020; Pietrofesa et al. 2017). Meanwhile, SDG can also activate the Nrf2 signaling pathway to exert cytoprotective effects, thereby inhibiting the accumulation of reactive oxygen species (ROS) (Pietrofesa et al. 2016). These properties fully highlight the potential value of SDG as a therapeutic agent for ALI. However, the specific role and underlying mechanisms of SDG in ALI remain unclear at present. Based on this, the present study established an LPS‐induced mouse model of ALI to investigate the effects of SDG on ALI and its related mechanisms. Additionally, an LPS‐induced inflammatory model using RAW264.7 mouse macrophages was constructed to analyze the regulatory effect of SDG on the inflammatory response of these cells in vitro.

## 2 Materials and Methods

### 2.1 Animals

Male C57BL/6 mice (age, 6–7 weeks; weight, 17–20 g) were housed under standard laboratory conditions at 25°C with 55% humidity and a 12 h light‐dark cycle prior to the start of the experiments. All mice were acclimated for 7 days before the initiation of the experiments. All experimental procedures were approved by the Ethics Committee of People's Hospital of Leshan (20230319). All methods were conducted in accordance with relevant guidelines and regulations, and in accordance with ARRIVE guidelines.

### 2.2 ALI Model and SDG Treatment Procedure

The mice were randomly divided into four groups (*n* = 6 in each group): control group, LPS group, L‐SDG group, and H‐SDG group. The LPS‐induced ALI mouse model was established as described in previous studies, which was done by intranasal administration of 10 μL of 4 mg/mL LPS solution (Sigma‐Aldrich, USA) (Tian et al. 2019; Szarka et al. 1997; Shokry et al. 2022). Starting from 7 days prior to LPS challenge, mice were administered SDG (MedChemExpress, USA) via gavage at doses of 50 and 100 mg/kg daily. This dosing regimen was based on previous experience in other rodent inflammatory models (Christofidou‐Solomidou et al. 2019). At 24 h after LPS exposure, the mice were anesthetized via intraperitoneal injection of ketamine (20 mg/kg) and xylazine (3 mg/kg). Bronchoalveolar lavage fluid (BALF) and lung tissues were then collected for further analysis. The mice were euthanized by CO_2_ asphyxia and subsequent cervical dislocation.

### 2.3 Cell Lines and Culture Conditions

Murine macrophage RAW264.7 cells were purchased from ATCC (TIB‐71) and cultured with DMEM Medium (Gibco, USA) containing 10% fetal bovine serum (FBS) (Gibco, USA), incubated in a humidified incubator containing 5% CO_2_ at 37°C. Cells were divided into four groups: the control group received 1% DMSO and PBS, the LPS group received 1% DMSO and LPS (100 ng/mL); l‐SDG group received 10 μM SDG and LPS (100 ng/mL); the H‐SDG group received 20 μM SDG and LPS (100 ng/mL) followed by incubation for 24 h (Z. Wang et al. 2020).

### 2.4 MCC950 Treatment in RAW264.7 Cells

RAW264.7 macrophages were seeded in six‐well plates and cultured overnight. Cells were pretreated with MCC950 (30 μM, MedChemExpress, USA) or vehicle for 6 h, then stimulated with LPS (100 ng/mL) with or without SDG (20 μM) for 24 h. After treatment, cell lysates were collected for Western blot analysis of NLRP3, cleaved caspase‐1, and GSDMD‐N; culture supernatants were harvested for IL‐1β and IL‐18 ELISA.

### 2.5 Hematoxylin and Eosin (H&E) Stain and Lung Injury Score

Mice were anesthetized with 1% pentobarbital sodium and painlessly sacrificed. Their lungs and heads were removed and fixed in 4% paraformaldehyde. The specimens were then decalcified in ethylenediaminetetraacetic acid (EDTA) decalcification solution and embedded in paraffin. The paraffin‐embedded tissue was sectioned anteroposteriorly at a thickness of 5 µm, deparaffinized in xylene, and rehydrated in ethanol. The sections were then stained with H&E for examination of the lung and nasal mucosa. Morphological changes in lung tissues and nasal mucosa were evaluated by light microscopy in a blinded fashion. The lung injury score was determined using the established scoring system as previously reported (Kulkarni et al. 2022).

### 2.6 Wet/Dry Weight Ratio (W/D)

The upper lobe of the right lung from each mouse was gently blotted with filter paper to remove surface fluids and blood, after which its wet weight was immediately recorded using an analytical balance. The tissue was then dehydrated at 80°C for 24 h in a drying oven to obtain the dry weight. The W/D was subsequently calculated to assess pulmonary edema severity (Lai et al. 2023).

### 2.7 BALF Collection and Measurement of Protein Concentration

BALF was collected by instilling 1 mL of sterile PBS into the lungs through an exposed tracheal opening. After three cycles of lavage and fluid recovery, the BALF was centrifuged at 800*g* for 10 min, and the supernatant was assessed for protein levels using a BCA protein assay kit (Beyotime, China).

### 2.8 Enzyme‐Linked Immunosorbent Assay (ELISA)

The lung tissues were homogenized and centrifuged to obtain supernatants. Superoxide dismutase (SOD) and catalase (CAT) activities, along with malondialdehyde (MDA) content, were determined using specific ELISA kits (Jiancheng, China). The concentrations of IL‐18, IL‐1β, and TNF‐α in the BALF and CCL2 in the lung were measured by using ELISA kits (CUSABIO, China, and R&D Systems, USA). All measurements were performed according to the manufacturer's instructions.

### 2.9 Immunohistochemistry (IHC) Analysis

Paraffin‐embedded lung tissue was sectioned into 5 µm slices. After antigen unmasking, 3% hydrogen peroxide was applied to block endogenous peroxidase activity for 20 min. Nonspecific binding was blocked with 5% bovine serum albumin (BSA) for 90 min, followed by incubation overnight at 4°C with NLRP3 antibody (1:1000, Proteintech, USA) and caspase‐1 antibody (1:1000, Abcam, USA). The following day, the sections were incubated with the corresponding secondary antibody for 60 min at room temperature, then stained with DAB solution at room temperature. All histological images were assessed by two independent investigators who were blinded to the study.

### 2.10 Cell Counting Kit‐8 (CCK‐8) Assay

The cytotoxic effects of SDG on RAW264.7 cells were evaluated using a CCK‐8 assay (Beyotime Biotechnology, China). Cells in the logarithmic growth phase were seeded into 96‐well plates at a density of 1 × 10^4^ cells per well and incubated overnight for attachment. The medium was then replaced with fresh medium containing various concentrations of SDG (0, 5, 10, 20, 40, 60, 100 µM) and incubated for 24 h. Following treatment, the supernatant was carefully aspirated, and 100 μL of DMEM containing 10% CCK‐8 was added to each well. After incubation at 37°C for 1 h, the optical density (OD) was measured at 450 nm using a microplate reader. Wells containing culture medium without cells were used as blanks, and cells treated with vehicle (0 µM) served as the control (defined as 100% viability). Cell viability was calculated using the following formula:

$$\text{Cell viability}(\%)=\frac{{\mathrm{OD}}_{\text{sample}}-{\mathrm{OD}}_{\text{blank}}}{{\mathrm{OD}}_{\text{control}}-{\mathrm{OD}}_{\text{blank}}}\times 100$$

### 2.11 Immunofluorescence Staining

Following fixation with 4% paraformaldehyde (15 min) and washed three times with PBS, cells were treated with 3% H₂O₂ and blocked with 5% BSA. Immunostaining was performed using primary antibodies against NLRP3 and caspase‐1 (1:500, Cell Signaling Technology, USA) at 4°C overnight, followed by 1 h room temperature incubation with Alexa Fluor 594‐conjugated secondary antibody. Nuclei were counterstained with DAPI‐containing mounting medium (ZSGB‐BIO, China), and images were acquired using a Leica DM6 fluorescence microscope.

### 2.12 Quantitative Real‐Time Polymerase Chain Reaction (qRT‐PCR)

Total RNA was extracted from the lung using TRIzol reagent (Invitrogen, USA), and reverse‐transcribed into cDNA using Transcriptor First Strand cDNA Synthesis Kit (Thermo Scientific, USA). Real‐time quantitative polymerase chain reaction (RT‐qPCR) analysis was performed using PowerUpTM SYBRTM Green Master Mix (Thermo Scientific, USA). The target gene mRNA expression was normalized with the housekeeping gene β‐actin, and the relative gene expression was calculated by $2^{-\Delta \Delta C_{t}}$. *IL‐1β, IL‐18, TNF‐α, CCL2*, and *β‐actin* primers used in this study were synthesized by Sangon Biotech (Shanghai, China). Primers used were as follows:: *IL‐1β*, 5′‐TCGCAGCAGCACATCAACAAGAG‐3′ (forward) and 5′‐AGGTCCACGGGAAAGACACAGG‐3′ (reverse); *IL‐18*, 5′‐CAAAGTGCCAGTGAACCCCAGAC‐3′ (forward) and 5′‐ACAGAGAGGGTCACAGCCAGTC‐3′ (reverse); *TNF‐α*, 5′‐CTGAACTTCGGGGTGATCGG‐3′ (forward) and 5′‐GGCTTGTCACTCGAATTTTGAGA‐3′ (reverse); *CCL2*, 5′‐GCTACAAGAGGATCACCAGCAG‐3′ (forward) and 5′‐GTCTGGACCCATTCCTTCTTGG (reverse); *β‐actin*, 5′‐GATGCTCCCCGGGCTGTATT‐3′ (forward) and 5′‐GGGGTACTTCAGGGTCAGGA (reverse).

### 2.13 Western Blot Analysis

Mice lung tissues were harvested and lysed in RIPA lysis buffer (Servicebio, China). The concentration of proteins was measured using a bicinchoninic acid protein assay kit (Servicebio, China). Total proteins were subjected to sodium dodecyl sulfate polyacrylamide gel electrophoresis (SDS‐PAGE) and then transferred to polyvinylidene fluoride (PVDF) membranes. The PVDF membranes were blocked with 5% fat‐free milk for 1 h. The membranes were incubated overnight at 4°C with the following primary antibodies: anti‐pp65 antibody (1:1000, Cell Signaling Technology, USA), anti‐p65 antibody (1:2000, Cell Signaling Technology, USA), anti‐NLRP3 antibody (1:1000, Cell Signaling Technology, USA), anti‐GSDMD‐N antibody (1:1000, Cell Signaling Technology, USA), anti‐caspase‐1 antibody (1:1000, Cell Signaling Technology, USA), anti‐β‐actin antibody (1:5000, Cell Signaling Technology, USA). On the following day, the PVDF membranes were washed with TBST and incubated with horseradish peroxidase (HRP)‐conjugated secondary antibodies for 1 h at room temperature. The bands were visualized using an enhanced chemiluminescence (ECL) solution. Western blot bands were analyzed using the mean grey value with ImageJ software and normalized to β‐actin expression.

### 2.14 Statistical Analysis

All data were analyzed using SPSS software (ver. 26.0). Data are expressed as mean ± standard error of the mean (SEM). Statistical comparisons were performed by using the Student's *t*‐test or one‐way ANOVA followed by Bonferroni correction. In all analyses, *p* < 0.05 was considered statistically significant.

## 3 Results

### 3.1 SDG Ameliorated LPS‐Induced ALI and Nasal Mucosal Damage

**Figure 1:**
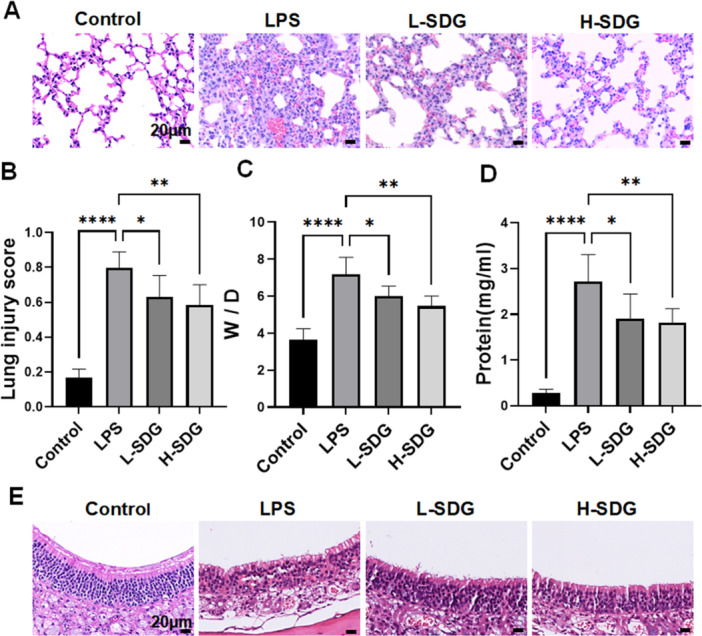
SDG ameliorated LPS‐induced ALI and nasal mucosal damage. (A, B) Representative H&E staining images of lung tissues and lung injury scores. Scale bar = 20 μm. (C) Lung W/D ratio. (D) Protein concentration in BALF. (E) Representative H&E staining images of nasal mucosa tissues. Scale bar = 20 μm. *n* = 6 mice per group. All data are presented as mean ± SEM. *p < 0.05, **p < 0.01, ***p < 0.001, ****p < 0.0001. BALF, bronchoalveolar lavage fluid; H&E, hematoxylin and eosin; W/D, wet/dry weight ratio.

To evaluate the protective effects of SDG against LPS‐induced ALI and identify its optimal therapeutic dosage, mice were treated with SDG before ALI was induced by intratracheal administration of LPS. As evidenced by lung histopathology, LPS challenge successfully induced acute lung tissue damage compared to the control group, as demonstrated by a significant increase in lung injury scores. The LPS‐induced pathology was characterized by pronounced tissue damage, extensive alveolar hemorrhage, marked thickening of alveolar walls, and inflammatory cell infiltration. Notably, both low‐ and high‐dose SDG treatments markedly reduced the lung injury scores and ameliorated these pathological changes in lung tissue (*p *< 0.01; Figure 1A,B). Compared to the control group, the LPS‐induced mice exhibited significantly increased lung W/D ratio and protein levels (*p *< 0.01; Figure 1C,D) indicating impairment of alveolar capillary barrier. Furthermore, intervention with either low‐ or high‐dose SDG restored these changes (*p* < 0.01; Figure 1C,D). Given that LPS were administered via intranasal instillation, we further investigated their potential effects on the nasal mucosa. Histopathological examination revealed that intranasal LPS challenge induced significant nasal mucosal damage, characterized by markedly increased inflammatory cell infiltration and epithelial cell injury compared to control mice. Notably, treatment with SDG significantly attenuated these pathological changes (Figure 1E). Collectively, these results suggested that low‐ or high‐dose SDG have protective effects against LPS‐induced ALI and nasal mucosal damage.

### 3.2 SDG Attenuated Oxidative Stress During LPS‐Induced ALI

**Figure 2:**
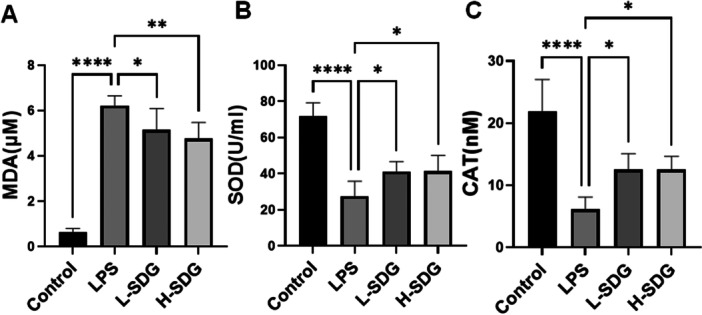
SDG attenuated oxidative stress during LPS‐induced ALI. (A) The level of MDA in the lung tissues was detected by ELISA. (B) The level of SOD in the lung tissues was detected by ELISA. (C) The level of CAT in the lung tissues was detected by ELISA. *n *= 6 mice per group. All data are presented as mean ± SEM. *p < 0.05, **p < 0.01, ***p < 0.001, ****p < 0.0001. CAT, catalase; MDA, malondialdehyde; SOD, superoxide dismutase.

Oxidative damage significantly exacerbates the pathological progression of ALI. Given that SDG possesses antioxidative properties, we investigated whether SDG treatment could mitigate LPS‐induced oxidative stress. Compared with the control group, the LPS group demonstrated elevated levels of oxidative stress markers MDA and reduced levels of antioxidant stress markers (SOD and CAT) in lung tissues (*p *< 0.01; Figure 2A−C). However, low‐ or high‐dose SDG treatment significantly reduced MDA level and increased SOD and CAT compared to the LPS‐challenged group (*p *< 0.05; Figure 2A−C). Taken together, these results demonstrated that SDG confers protection against LPS‐induced ALI partly through attenuation of oxidative stress pathways.

### 3.3 SDG Alleviated Inflammatory Responses and Macrophage Infiltration

**Figure 3:**
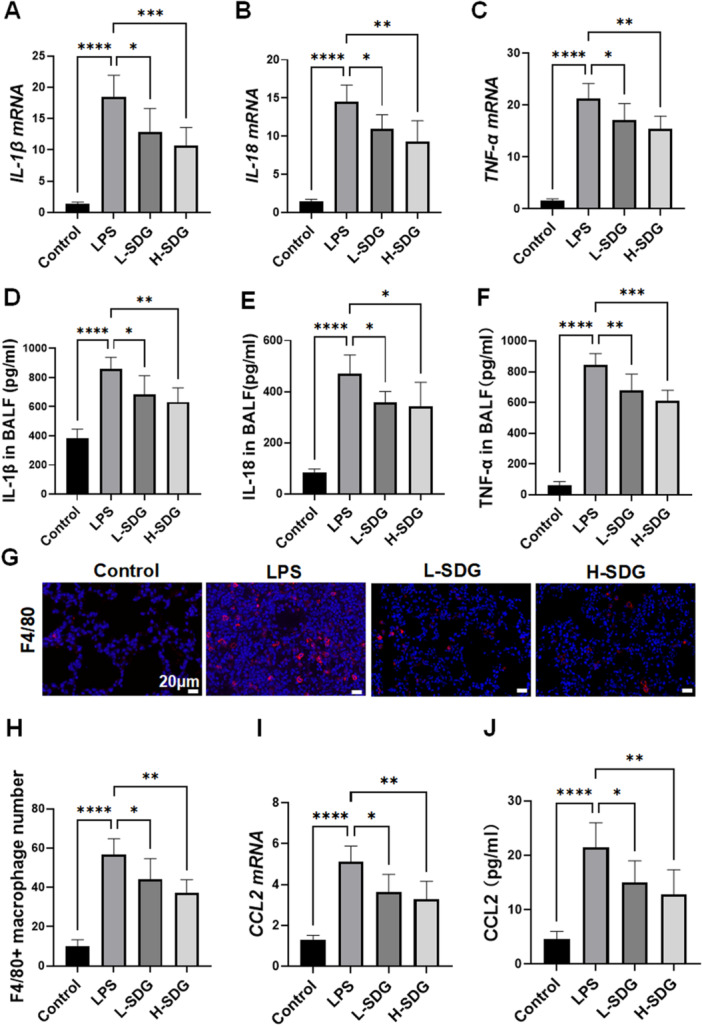
SDG alleviated inflammatory responses and macrophage infiltration. The mRNA levels of *IL‐1β* in lung tissues by qRT‐PCR. (B) The mRNA levels of *IL‐18* in lung tissues by qRT‐PCR. (C) The mRNA levels of *TNF‐α* in lung tissues by qRT‐PCR. (D) The level of IL‐1β in BLAF was detected by ELISA. (E) The level of IL‐18 in BLAF was detected by ELISA. (F) The level of TNF‐α in BLAF was detected by ELISA. (G) Immunofluorescence staining of F4/80 (red) and DAPI (blue) in lung tissues. DAPI stains the nuclei. Scale bar = 20 μm. (H) Number of F4/80+ cells. Quantification of cells in the field of view. (I) The mRNA levels of *CCL2* in lung tissues by qRT‐PCR. (J) The level of CCL2 in lung tissues was detected by ELISA. *n* = 6 mice per group. All data are presented as mean ± SEM. *p < 0.05, **p < 0.01, ***p < 0.001, ****p < 0.0001.

To further investigate the anti‐inflammatory effects of SDG, we measured the gene expression and secretion of pro‐inflammatory cytokines IL‐1β, IL‐18, and TNF‐α, which are key mediators of LPS‐induced inflammation. Compared to the control group, LPS challenge significantly upregulated both gene expression and secretion of IL‐1β, IL‐18, and TNF‐α (*p *< 0.01; Figure 3A−F). However, SDG treatment effectively counteracted these changes in mice, downregulating the expression and release of these inflammatory mediators (*p *< 0.05; Figure 3A−F). Given that pro‐inflammatory cytokines (TNF‐α and IL‐1β) are primarily secreted by macrophages, we further investigated the alterations in pulmonary macrophages. Immunofluorescence staining of lung tissues revealed significantly increased infiltration of F4/80⁺ macrophages in ALI mice compared to the control group, accompanied by elevated CCL2 gene expression and protein content (*p *< 0.01; Figure 3G−J). SDG treatment markedly attenuated these changes, reducing macrophage infiltration and downregulating both CCL2 mRNA levels and protein secretion (*p *< 0.05; Figure 3G−J). These observations suggested that SDG exerted anti‐inflammatory effects and reduced macrophage infiltration, at least partially through suppression of CCL2 expression.

### 3.4 SDG Inhibited NF‐κB/NLRP3 Pathway in ALI

**Figure 4:**
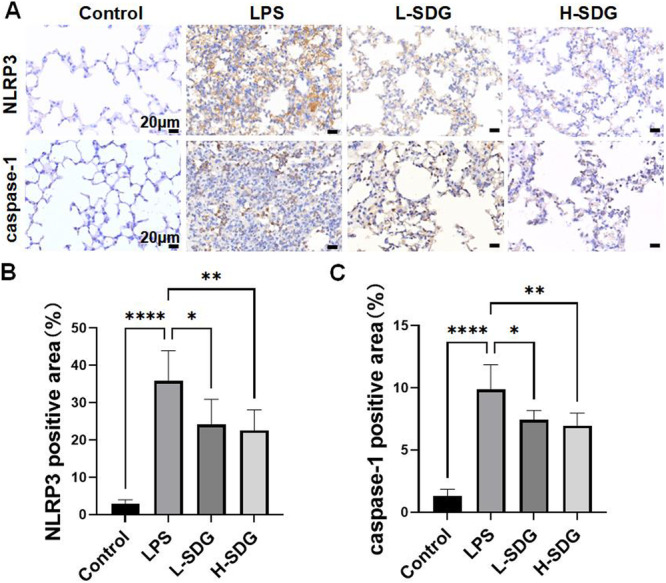
SDG decreased the expression of NLRP3 and caspase‐1 in lung tissues. (A) Representative IHC images showing the expression of NLRP3 in the lung tissues. Scale bar = 20 μm. (B) Representative IHC images showing the expression of caspase‐1 in the lung tissues. Scale bar = 20 μm. (C) Quantitation of NLRP3‐positive area. (D) Quantitation of caspase‐1 positive area. *n* = 6 mice per group. All data are presented as mean ± SEM. *p < 0.05, **p < 0.01, ***p < 0.001, ****p < 0.0001.

**Figure 5:**
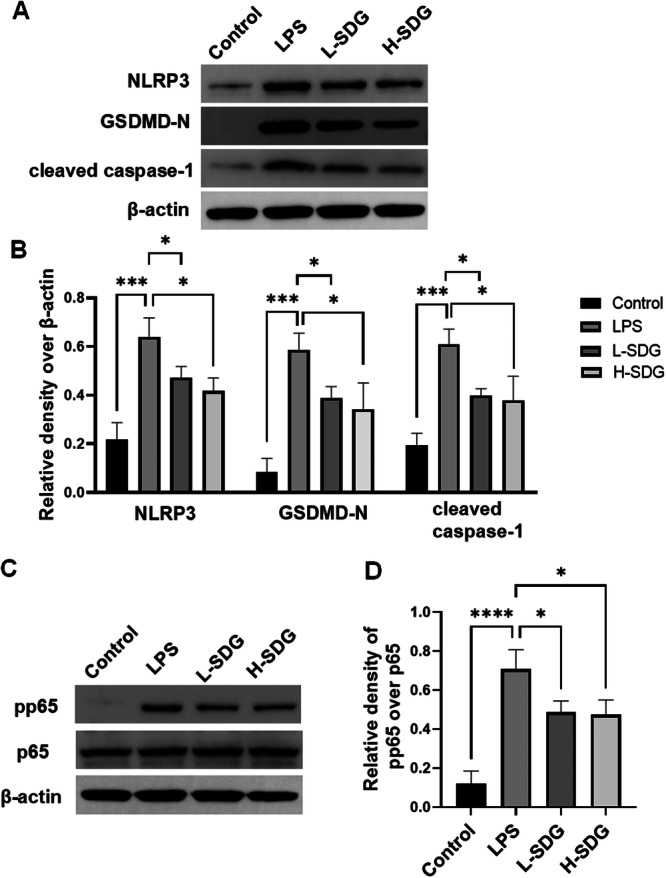
SDG suppressed the NF‐κB/NLRP3 pathway in lung tissues. (A) Western blot analysis of protein levels of NLRP3, GSDMD‐N, cleaved caspase‐1, and β‐actin. (B) Quantification of NLRP3, GSDMD‐N, and cleaved caspase‐1, with β‐actin used as a loading control. (C) Western blot analysis of protein levels of pp65, p65, and β‐actin. (D) Quantification of pp65 over p65. *n* = 3 mice per group. All data are presented as mean ± SEM. *p < 0.05, **p < 0.01, ***p < 0.001, ****p < 0.0001.

Our study revealed that LPS‐induced ALI significantly enhanced the expression of NLRP3 inflammasome‐activated cytokines IL‐1β and IL‐18, while SDG treatment effectively suppressed their production. These findings prompted us to further investigate whether SDG could inhibit NLRP3 inflammasome activation during ALI pathogenesis by IHC and western blot analysis. Compared with the control group, the positive expressions of NLRP3 and caspase‐1 in the LPS group significantly increased (*p *< 0.01; Figure 4A−C). These effects were significantly reversed after treatment of SDG (*p *< 0.05; Figure 4A−C). Meanwhile, LPS exposure caused a significant increase in the protein levels of NLRP3, GSDMD‐N, and cleaved caspase‐1 in lung tissues compared to the control group (*p *< 0.01; Figure 5A,B). SDG treatment could significantly reduce the protein levels of NLRP3, GSDMD‐N, and cleaved caspase‐1 in ALI mice (*p *< 0.05; Figure 5A,B). As a master regulator of innate immunity and inflammation, the NF‐κB signaling pathway controls the transcription of proinflammatory cytokines during NLRP3 inflammasome activation. To elucidate the potential mechanism of SDG in ALI, we investigated its effects on both NF‐κB p65 and phospho‐p65. Compared with the control group, the protein expressions of pp65 in the LPS group markedly increased (*p *< 0.01; Figure 5C,D). However, NF‐κB p‐p65 were downregulated in ALI by SDG treatment (*p *< 0.05; Figure 5C,D). These results suggested that SDG inhibited the NF‐κB/NLRP3 signaling pathway.

### 3.5 SDG Suppressed NF‐κB/NLRP3 Pathway in RAW264.7

**Figure 6:**
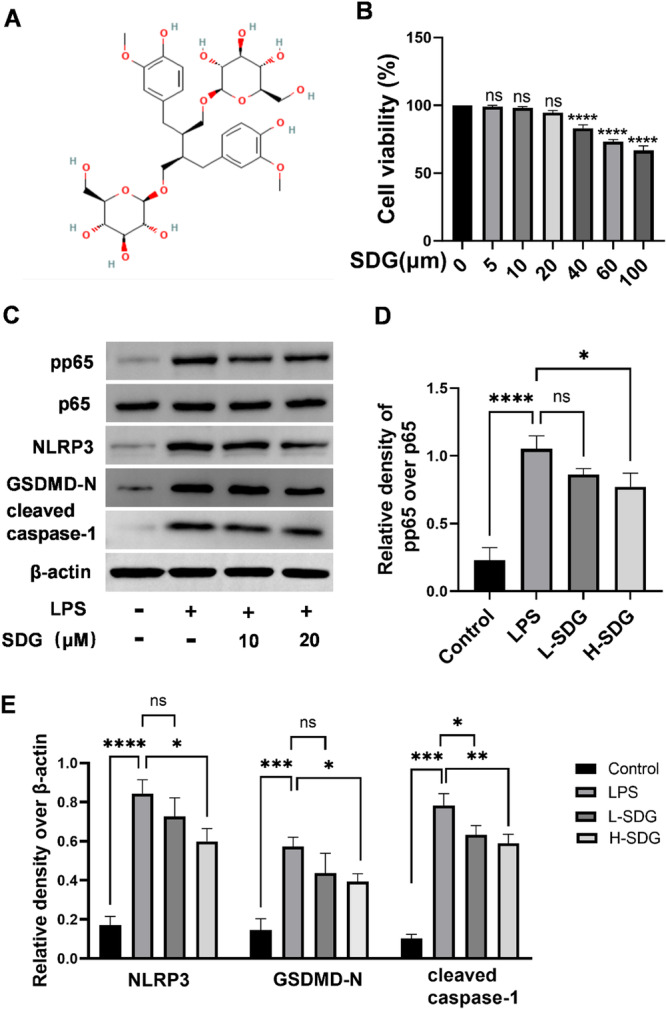
SDG suppressed NF‐κB/NLRP3 pathway in RAW264.7 cells. (A) Chemical structure of SDG. (B) Viability of RAW264.7 cells after 24 h treatment with SDG (0–100 µM). *p < 0.05, **p < 0.01, ***p < 0.001, ****p < 0.0001 compared to the control group (0 µM). (C) Western blot analysis of protein levels of pp65, p65, NLRP3, GSDMD‐N, cleaved caspase‐1, and β‐actin in RAW264.7 cells. (D) Quantification of pp65 over p65. (E) Quantification of NLRP3, GSDMD‐N, and cleaved caspase‐1, with β‐actin used as a loading control. *n* = 3 per group. All data are presented as mean ± SEM. *p < 0.05, **p < 0.01, ***p < 0.001, ****p < 0.0001. ns, no significance.

**Figure 7:**
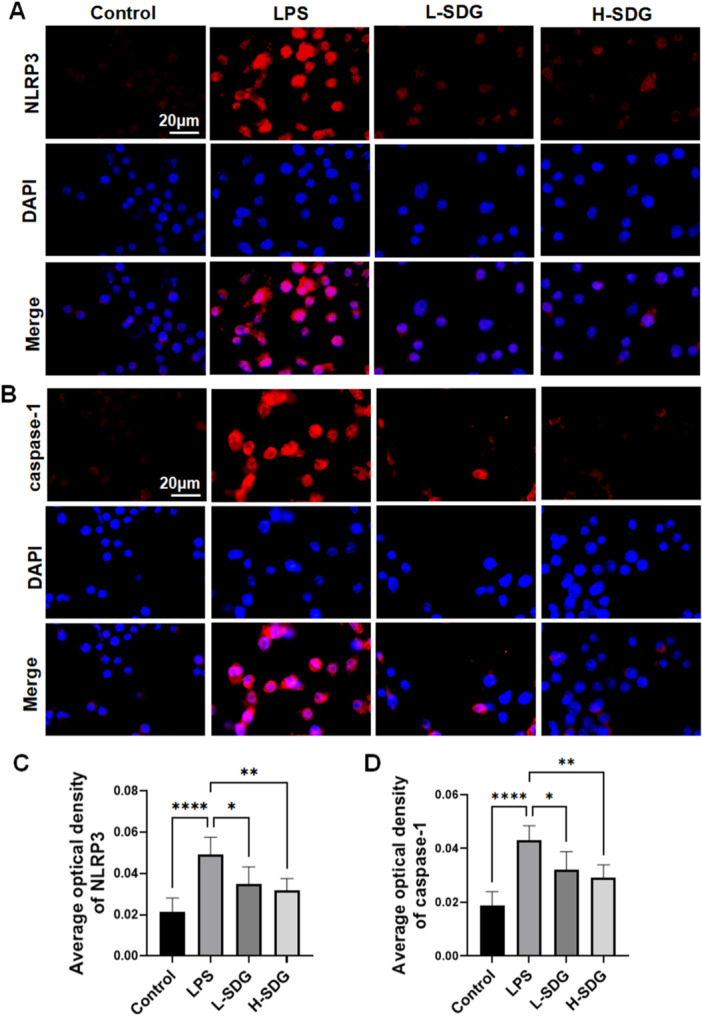
SDG decreased the expression of NLRP3 and caspase‐1 in RAW264.7 cells. (A) Immunofluorescence staining of NLRP3 (red) and DAPI (blue) in RAW264.7. DAPI stains the nuclei. Scale bar = 20 μm. (B) Immunofluorescence staining of caspase‐1 (red) and DAPI (blue) in RAW264.7. DAPI stains the nuclei. Scale bar = 20 μm. (C) Quantitation of NLRP3 average optical density. (D) Quantitation of caspase‐1 average optical density. *n* = 6 per group. All data are presented as mean ± SEM. *p < 0.05, **p < 0.01, ***p < 0.001, ****p < 0.0001.

To further investigate the therapeutic mechanisms of SDG in ALI, we employed an in vitro inflammation model using LPS‐stimulated RAW264.7. First, we assessed the cytotoxicity of SDG in RAW264.7 cells using a CCK‐8 assay. The results showed that SDG did not significantly affect cell viability at concentrations below 20 µM (*p *> 0.05; Figure 6B). However, a significant and dose‐dependent reduction in cell viability was observed starting at 40 µM (*p *< 0.05; Figure 6B). Therefore, SDG concentrations of 10 and 20 µM were selected for subsequent in vitro experiments to represent low and high doses, respectively. To determine whether the NF‐κB/NLRP3 pathway alterations observed in vivo were recapitulated in vitro, we performed western blot analysis using LPS‐stimulated RAW264.7 macrophages. LPS stimulation markedly upregulated the protein levels of pp65, NLRP3, GSDMD‐N, and cleaved caspase‐1 in RAW264.7 compared to the control group (*p *< 0.01; Figure 6C−E). SDG treatment effectively reversed these changes, reducing expression levels of pp65, NLRP3, GSDMD‐N, and cleaved caspase‐1 (*p *< 0.05; Figure 6C−E). Immunofluorescence analysis revealed that LPS challenge significantly enhanced the fluorescence intensity of both NLRP3 and caspase‐1 compared to control groups (*p *< 0.01; Figure 7A−C). SDG treatment markedly attenuated these LPS‐induced effects, reducing NLRP3 and caspase‐1 fluorescence intensity (*p *< 0.05; Figure 7A−C). These data demonstrated that SDG exerts protective effects in LPS‐stimulated RAW264.7 macrophages by modulating the NF‐κB/NLRP3 signaling pathway.

### 3.6 SDG Inhibited NLRP3 Inflammasome Activation in RAW264.7 Cells

**Figure 8:**
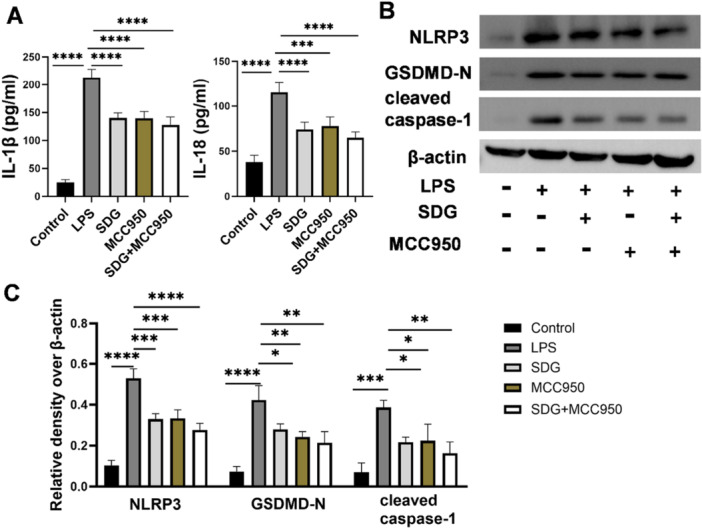
SDG inhibited NLRP3 inflammasome activation in RAW264.7 cells. (A) Levels of IL‐1β and IL‐18 in the culture supernatant of RAW264.7 cells. (B) Western blot analysis of protein levels of NLRP3, GSDMD‐N, cleaved caspase‐1, and β‐actin in RAW264.7 cells. (C) Quantification of NLRP3, GSDMD‐N, and cleaved caspase‐1, with β‐actin used as a loading control. *n* = 3 per group. All data are presented as mean ± SEM. *p < 0.05, **p < 0.01, ***p < 0.001, ****p < 0.0001.

To determine whether NLRP3 is a direct target of SDG, we employed MCC950, a specific NLRP3 inflammasome inhibitor, as a positive control in LPS‐stimulated RAW264.7 macrophages. MCC950 treatment significantly attenuated LPS‐induced IL‐1β and IL‐18 secretion, mirroring the effect of SDG treatment (*p *< 0.01; Figure 8A). Notably, combined treatment with SDG and MCC950 did not produce additive inhibitory effects on these cytokines compared to either agent alone (*p *> 0.05; Figure 8A), suggesting they act on the same pathway. Western blot analysis demonstrated that both SDG and MCC950 significantly inhibited the LPS‐induced upregulation of NLRP3, GSDMD‐N, and cleaved caspase‐1 (*p *< 0.05; Figure 8B,C). Importantly, combined treatment with SDG and MCC950 did not result in additive inhibitory effects compared to either agent alone (*p *> 0.05; Figure 8B,C). Together, these results demonstrated that SDG exerted its protective effects primarily through inhibition of the NLRP3 inflammasome.

## 4 Discussion

This study systematically evaluated the protective effects of SDG against LPS‐induced ALI and its underlying mechanisms through in vivo and in vitro experiments. In the vivo study, administration of SDG significantly ameliorated LPS‐induced lung histopathological and functional impairments, inhibited pulmonary inflammation, reduced oxidative stress, and decreased macrophage infiltration. These protective effects were closely associated with modulation of the NF‐κB/NLRP3 signaling pathway. Furthermore, these findings were corroborated in an in vitro model using LPS‐stimulated RAW264.7 macrophages, demonstrating that SDG exerted direct effects on macrophages by suppressing the NF‐κB/NLRP3 signaling pathway, thereby mediating its protective actions.

In the present study, it was found that SDG significantly reduced the infiltration of F4/80⁺ macrophages in the lung tissues of ALI mice and downregulated the expression of the chemokine CCL2. This result provided a key clue for elucidating the mechanism underlying the effect of SDG in alleviating lung inflammation in ALI. During the pathological progression of ALI, macrophages serve as core effector cells (Cai et al. 2025). Their excessive recruitment and activation can release a large number of pro‐inflammatory cytokines, such as TNF‐α and IL‐6, which exacerbate the pulmonary inflammatory cascade. In severe cases, this even induces a cytokine storm, further aggravating lung tissue damage (J. W. Lee et al. 2021; Y. Wang et al. 2022). As a key chemokine mediating the migration of monocytes/macrophages, CCL2 can specifically bind to its receptor CCR2 to guide the aggregation of immune cells to the pulmonary inflammatory site, and it is an important signaling molecule for maintaining the continuous infiltration of macrophages in the lungs (Singh et al. 2021; Miyamoto et al. 2022). The inhibitory effect of SDG on CCL2 observed in this study can reduce the migration and infiltration of macrophages into lung tissues, thereby decreasing the aggregation of inflammatory effector cells at the site of lung injury. Ultimately, this exerted an effect of alleviating lung inflammation in ALI. This finding not only enriched the theoretical research on the anti‐inflammatory mechanism of SDG but also provided a new target direction for ALI treatment.

The NF‐κB/NLRP3 signaling pathway plays a crucial pathogenic role in multiple diseases, including pulmonary fibrosis, heart failure, and chronic colitis (D. Wang et al. 2024; Y. Zhang et al. 2022). The NF‐κB/NLRP3 pathway activation involves two‐stage process, priming and activation. In the priming phase, DAMP‐mediated stimulation of NF‐κB signaling upregulates expression of pro‐IL‐1β, pro‐IL‐18, NLRP3, ASC, and procaspase‐1. Subsequent activation signals induce NLRP3 inflammasome complex formation, which subsequently induces the splicing of procaspase‐1 itself to obtain activated caspase‐1 responsible for cleaving pro‐IL‐1β and pro‐IL‐18 into the mature and secreted forms IL‐1β and IL‐18 (Hooftman et al. 2020; Tang et al. 2023). These cytokines initiate a cascade of inflammatory responses that further amplify through autocrine activation, promoting monocyte/macrophage recruitment and exacerbating pulmonary inflammation. Secreted IL‐1β and IL‐18 induce a cascade of inflammatory responses through self‐activation, enhancing monocytes/macrophages recruitment and vascular inflammatory responses that subsequently reinforce NF‐κB/NLRP3 pathway activation (Z. Li et al. 2023). Previous studies have demonstrated that miRNA‐206‐3p alleviates ALI by suppressing NF‐κB/NLRP3 signaling, thereby inhibiting inflammasome assembly and subsequent cleavage of pro‐IL‐1β/pro‐IL‐18 (Chen et al. 2024). NF‐κB activation facilitates the transcription of NLRP3, pro‐IL‐1β, and pro‐IL‐18, thereby providing essential molecular components for inflammasome assembly (Kaszycki and Kim 2025) but a recent study found that NF‐κB restricted NLRP3 inflammasome activation via elimination of damaged mitochondria (Zhong et al. 2016). Our findings demonstrated that intranasal LPS administration significantly upregulated NF‐κB/NLRP3 pathway‐associated proteins in lung tissues, indicating NF‐κB‐mediated promotion of NLRP3 activation. Through both in vivo and in vitro experiments, we observed that SDG effectively suppressed LPS‐induced activation of the NF‐κB/NLRP3 pathway, thereby ameliorating ALI. Existing studies have confirmed that SDG can regulate pathological processes such as inflammatory response and oxidative stress by inhibiting the NF‐κB pathway; meanwhile, SDG can also downregulate the expression levels of NLRP1 and NLRP3—these findings are highly consistent with the results of the present study (Pietrofesa et al. 2017; Z. Wang et al. 2020; Bowers et al. 2019).

In summary, our research data demonstrated that SDG significantly alleviated ALI, reduced pathological lung tissue damage, inhibited oxidative stress and inflammatory responses, and decreased macrophage infiltration. These protective effects were closely associated with the downregulation of the NF‐κB/NLRP3 signaling pathway. This provided new insights into potential therapeutic targets for ALI and suggested that SDG may emerge as a promising drug for ALI treatment. Therefore, further clinical investigations were warranted to elucidate the therapeutic potential of SDG.

## Author Contributions

Z.W designed and wrote the paper. Yu.Z., Ya.Z., Z.Q., K.Z., H.L., J.Y., C.T., and Y.X. performed research. All authors have read and approved the final manuscript.

## Conflicts of Interest

The authors declare no conflicts of interest.

## Data Availability

The data sets supporting this study are available from the corresponding author (E‐mail address: sciwangzhen@163.com) upon justified request.
